# Impact of the COVID-19 pandemic on cervical cancer screening participation, abnormal cytology prevalence and screening interval in Catalonia

**DOI:** 10.3389/fonc.2024.1338859

**Published:** 2024-05-29

**Authors:** Claudia Robles, Laura Monfil, Raquel Ibáñez, Esther Roura, Rebeca Font, Paula Peremiquel-Trillas, Maria Brotons, Cristina Martínez-Bueno, Silvia de Sanjosé, Josep Alfons Espinàs, Laia Bruni

**Affiliations:** ^1^ Unit of Infections and Cancer – Information and Interventions, Cancer Epidemiology Research Programme, Institut Català d’Oncologia (ICO), Infections and Cancer Group, Epidemiology, Public Health, Cancer Prevention and Palliative Care Program, Institut d’Investigació Biomèdica de Bellvitge (IDIBELL), L’Hospitalet de Llobregat, Barcelona, Spain; ^2^ Centro de Investigación Biomédica en Red de Epidemiología y Salud Pública (CIBERESP - CB06/02/0073), Madrid, Spain; ^3^ Catalan Cancer Strategy, Department of Health, Catalonia, Spain; ^4^ University of Barcelona, Barcelona, Spain; ^5^ Atenció a la Salut Sexual i Reproductiva (ASSIR) Catalunya, Institut Català de la Salut, Barcelona, Spain; ^6^ Grup de Recerca en Salut Sexual i Reproductiva (GRASSIR), Barcelona, Spain; ^7^ Division of Cancer Epidemiology and Genetics, National Cancer Institute, Rockville, MD, United States; ^8^ Institut de Salut Global (ISGlobal), Barcelona, Spain

**Keywords:** cervical cancer, cancer screening program, Catalonia (NE Spain), COVID-19, impact, participation, abnormal cytology prevalence, screening interval

## Abstract

**Background:**

The COVID-19 pandemic led to a national lockdown and the interruption of all cancer preventive services, including cervical cancer screening. We aimed to assess the COVID-19 pandemic impact on opportunistic screening participation, abnormal cytology (ASCUS+) prevalence and screening interval in 2020 and 2021 within the Public Health System of Catalonia, Spain.

**Methods:**

Individual data on cytology and HPV testing of women aged 25–65 from 2014 to 2021 were retrieved from the Information System for Primary Care Services (SISAP). Time-series regression models were used to estimate expected screening participation and abnormal cytology prevalence in 2020 and 2021. The impact was determined by comparing observed and expected values (ratios). Additionally, changes in screening interval trends between 2014 and 2021 were assessed by fitting a Piecewise linear regression model.

**Results:**

Cervical cancer screening participation decreased by 38.8% and 2.2% in 2020 and 2021, respectively, with the most significant impact on participation (-96.1%) occurring in April 2020. Among older women, participation was lower, and it took longer to recover. Abnormal cytology prevalence was 1.4 times higher than expected in 2020 and 2021, with variations by age (range=1.1–1.5). From June 2020 onwards, the screening interval trend significantly changed from an increase of 0.59 to 3.57 months per year, resulting in a median time of 48 months by December 2021.

**Conclusions:**

During the pandemic, fewer women have participated in cervical cancer screening, abnormal cytology prevalence has increased, and the screening interval is more prolonged than before. The potential cervical cancer lifetime risk implications highlight the need for organized HPV-based screening.

## Introduction

The COVID-19 pandemic has had a significant impact on cancer screening programs around the world. Numerous countries worldwide have reported varying degrees of reductions in participation (1–5). For example, in comparison to 2019, Slovenia saw a 92% decline in cytology volume between March and May 2020 (6), while Belgium experienced a 43% reduction in volume between January and April 2020 (7). Meanwhile, the United States reported a 92% decrease in participation compared to 2019 during March and April 2020 (8). In Peru, a 76% reduction in cytology volume was observed for 2020 compared to the expected volume based on modeled data (9)

In Spain, a nationwide lockdown was imposed on March 14th 2020. All cancer preventive services, including cervical cancer screening, were suspended, and only follow-up appointments for previous abnormal results were maintained (10). The lockdown was gradually lifted between May 11^th^ and June 2020 (11), and primary screening in Catalonia was partially resumed in July 2020. In the two years since the pandemic began, six COVID-19 waves have occurred. Containment measures for these waves have varied across and within Spanish regions, including social distancing or mobility restriction within residential areas, although exceptions were made for medical visits.

The medium- and long-term impact of these disruptions on cervical cancer is still uncertain although Mexico has observed an increasing trend in the proportion of advanced stage and metastatic cancer cases (12). In Catalonia, the COVID-19 pandemic and related control measures have resulted in cancer underdiagnosis (13–15). In the two years since the lockdown, 11% fewer cervical cancers have been diagnosed, and as of February 2022, cervical cancer detection in Catalonia had not yet returned to pre-pandemic levels (15). The potential impact on the cancer stage is unknown. However, no differences have been observed in the prevalence of cancer diagnoses in pathology records from 2019 and 2020 in two hospitals in Girona (16).

When the pandemic began, Catalonia was offering opportunistic cytology-based cervical cancer screening to women aged 25 to 65 through the public primary care system. The first pandemic wave hit when the transition from cytology-based to HPV-based screening on clinician-collected samples had just begun through a pre-scale-up pilot in an area of Barcelona city on October 2019 (17). Therefore, a significant consequence of the pandemic in Catalonia was the interruption and delay in the transition to primary HPV testing that was underway. To date, HPV testing has not yet been extended to the entire territory due to the pandemic, which prompted the development of a newly organized cervical cancer screening program based on self-sampling. This new strategy aimed to counteract the reduced number of scheduled visits to facilitate social distancing and the lower willingness of potential participants to visit a health center after the lockdown period. Accordingly, a pilot program for HPV testing of self-collected samples was launched in July 2021 in Metropolitana Sud, a metropolitan area in the south of Barcelona city (18).

A better understanding of the post-lockdown screening landscape can help inform decisions on the best catch-up strategies for under screened women. However, only some studies have explored the impact of the pandemic beyond the lockdown period. Germany reported a lower average number of women screened per clinic in 2021 than in 2020 (19). In the United States, despite a rebound in the third quarter of 2020, a slow decline in screening rates has been observed up to the last quarter of 2021 (20).

The pandemic has likely led to longer screening intervals between cervical cancer screenings, as observed for breast and colorectal cancer screening in Belgium (21). However, the same study also found that there were either no differences or shorter screening intervals in cervical cancer screening during the pandemic compared to before, probably due to the opportunistic screening and follow-up tests done during the lockdown. Women with longer screening intervals have a higher risk of disease and therefore higher prevalences of abnormal cytology or biopsy results are expected. In Brazil, the prevalence of abnormal cytology findings remained unchanged overtime; however, in 2020 and 2021, the CIN3 prevalence doubled compared to the 1.9% observed in 2019 (22). Other studies have only explored changes in the number of cases, and as observed with cancer diagnoses, underdiagnosis of high-grade cytologies and CIN2+ cases have been described in Slovenia and Canada, respectively (6, 23).

We evaluated the impact of the COVID-19 pandemic on opportunistic screening participation, abnormal cytology prevalence, and screening intervals within the Catalan public health system throughout the first two years after the pandemic started (period 2020–2021) using primary care electronic health records data.

## Materials and methods

We performed a time-series analysis of 2020 and 2021 on the participation, abnormal cytology prevalence and screening interval in the opportunistic cervical cancer screening program of Catalonia.

### Study population

Catalonia is a northeastern region of Spain with a total population of almost 8 million, including about 2.2 million women aged between 25–65 years who are targeted for cervical cancer screening.

In Catalonia, screening is opportunistic and free of charge within the public health system, which is administratively divided into seven health regions. Women are screened based on their age and previous screening history, either when they request it or when they visit the sexual and reproductive health centers for other reasons. At the time of this analysis, screening is still mainly cytology-based, with repeat testing every three years, except in two piloting areas where cytology is offered only to women aged 25–29 years, and HPV testing is being implemented every five years to those aged 30–65 years. The scale-up to a full organized HPV-based program is being planned for the coming years.

### Data source

Anonymized individual data were retrieved from the Primary Care Services Information System (SISAP). This is an electronic healthcare database containing structured primary care data from the Catalan Health Institute (ICS), the leading primary care provider of public health services in Catalonia. This provider manages about 75% of all Catalan primary care practices and covers around 80% of the Catalan population, which has proven to be highly representative of the population of Catalonia in terms of geographical area, age distribution and gender (24).

Historical screening self-reported or systematically registered data from 2008 onwards were retrieved from all women aged 25–65 who were assigned to primary care centers under the ICS between 2014 and 2021 regardless of their cervical cancer or hysterectomy history. Data included demographics (age and administrative health region) and all recorded cervical cancer screening related data, including the sampling date, type of screening test (cytology, HPV testing or both) and test results.

### Statistical analysis

The impact of the pandemic was explored on screening participation (defined as the proportion of screened women among the number of age-eligible women), abnormal cytology prevalence (defined as the proportion of cytologies with a result of atypical squamous cells of undetermined significance or worse (ASC-US+) among the total number of cytologies performed with a valid result), and screening interval (defined as the median number of months between the current screening event and the last screening cytology among women with no history of abnormal results).

Time-series regression was used to estimate the monthly expected screening participation and the abnormal cytology prevalence for 2020 and 2021.

Data from 2014 to 2018 (training set) were used to initially forecast the expected estimates for 2019 (test set) to validate the model. Data decomposition by moving averages showed a trend and seasonality pattern. Therefore, potentially suitable predictive models included a i) time series linear model potentially adjusted for trend and seasonality, ii) an autoregressive integrated moving average (ARIMA) model and iii) a Holt-winters additive model (25–27). Among these, the best-fit model was selected based on performance metrics (lower errors), residual examination and forecast values in the overall population. Further details on the model validation and selection are provided in **Supplementary Material 1**.

Once the best model was defined, 2019 data was incorporated into the regression models to project a 24-month forecast of the screening participation and abnormal cytology prevalence along with their 95% prediction interval (95%PI) over 2020 and 2021. The administrative health region of Girona (only 3.3% of the analysis population) had to be excluded from the forecast models for the overall Catalonia and age-stratified estimates because it behaved as an outlier and produced biased results. It is a region where the COVID-19 containment measures and the resumption of screening activity differed significantly from the rest of the regions (early 2022 instead of summer 2020 elsewhere in Catalonia).

Best-fit models were a time-series regression model adjusted for trend and seasonality for the screening participation analysis, whereas the expected abnormal cytology prevalence was estimated by adjusting a Holt-winters model.

The monthly impacts of COVID-19 on screening participation and abnormal cytology prevalence were estimated as ratios of observed values to expected ones. Ratio ranges included the ratios obtained when observed estimates are compared to the lower and the upper bounds of the 95%PI expected values. Expected values for time periods were estimated as the sum of monthly expected values except for those months in which expected values were not statistically different from observed ones, in which case, the latter were used.

Changes in abnormal cytology prevalence may result from increased screening intervals due to lower participation in screening. Therefore, the median number of months since the last screening cytology (not follow-up) with a normal result was estimated for all women with no history of previous abnormal results, screened between 2014 and 2021 and irrespective of the screening test used. The screening intervals’ median trend over time was assessed by fitting a piecewise linear regression model to identify potential breakpoints (changes in trend) and to quantify the increases in the median time for each identified period. Differences by abnormal cytology status were assessed using a non-parametric equality-of-medians test.

Analyses were conducted using statistical R version 4.2.1 (http://www.R-project.org) and Stata version 16.1.

### Ethical aspects

This research has been approved by the Ethics Committee from the Hospital Universitari de Bellvitge (code PR271/11). It complies with the national and international regulations on ethics and data protection.

## Results

### Screening participation

Overall, 1,598,069 women aged 25–65 years were assigned to an ICS primary care and ICS gynecology service between 2014 and 2021. Out of these, 706,432 women participated in cervical cancer screening at least once during this period. The average monthly screening participation between 2014 and 2019 was 0.88%, with lower participation in August and December (summer and Christmas holidays).

Time-regression analyses showed that in 2020 there was a reduction in participation of 38.8% (ratio=0.61; range=0.56,0.68) in women aged 25–65 years (**Table 1**). The predicted monthly screening participation in 2020 based on previous years would have ranged between 0.53 and 1.05% of eligible women aged 25–65. However, observed participation dropped to 0.04% of eligible women screened in April 2020 (ratio=0.04; range=0.03,0.05) (**Supplementary Table 1**). Over the full year 2021, screening participation was reduced by 2.2% (ratio=0.98; range=0.97,0.99) due to the 24% lower participation observed in January 2021 (ratio=0.76; range=0.66,0.89).

**Table 1:** Participation ratio between observed and expected participation by age group in 2020–2021.

Age group	N	2020			N	2021		
		Expected participation (%)	Observed participation (%)	Participation ratio (range)		Expected participation (%)	Observed participation (%)	Participation ratio (range)
**25–65**	1,371,549	10.94	6.70	0.61 (0.56,0.68)	1,370,417	10.79	10.56	0.98 (0.97,0.99)
**25–34**	285,544	13.53	8.90	0.66 (0.61,0.72)	280,163	13.89	13.50	0.97 (0.95,0.99)
**35–44**	374,663	12.00	7.52	0.63 (0.57,0.7)	363,514	11.98	11.76	0.98 (0.97,0.99)
**45–54**	378,690	10.28	6.12	0.60 (0.53,0.68)	386,099	10.17	9.95	0.98 (0.96,0.99)
**55–65**	332,651	8.18	4.53	0.55 (0.49,0.63)	340,641	7.96	7.56	0.95 (0.92,0.98)

The range shows the proportional change of the observed participation versus the 95% prediction interval of the expected participation.

**Figure 1:**
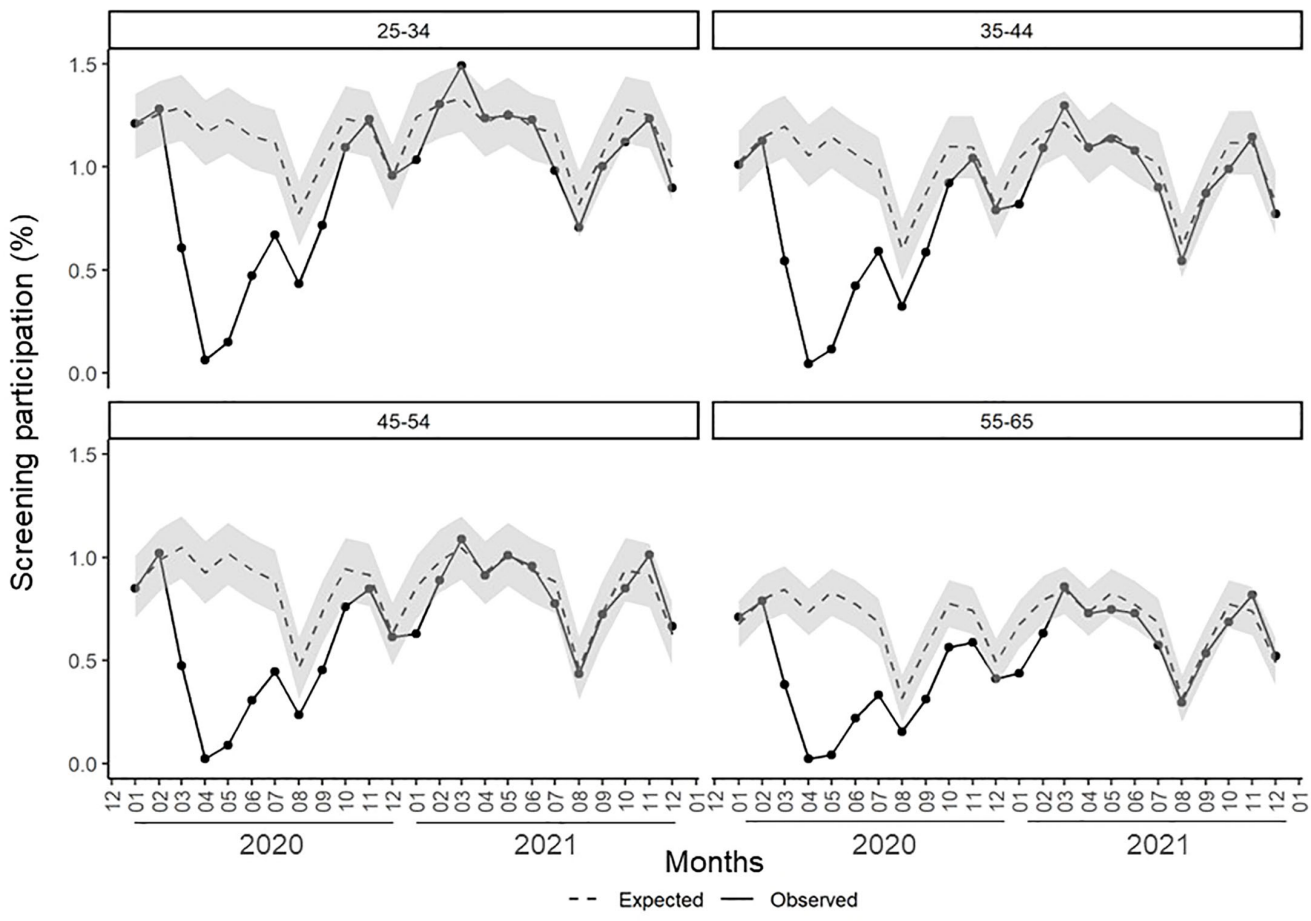
Monthly screening participation (expected and observed) in 2020 and 2021 in Catalonia by age group. Grey area depicts the 95% prediction interval of the expected monthly participation based on observed data from 2014-2019.

Stratified results by age groups (**Table 1** and **Figure 1**) showed higher reductions in observed versus expected participation at increasing age groups (in 2020, 34.3% fewer women aged 25–34 were screened compared to 44.7% fewer women aged 55–65). Expected screening participation estimates were achieved at a different pace depending on the age group (**Supplementary Table 2**); in women aged 25–34, participation recovery was achieved in October 2020; in women aged 35–44 and 45–54 years, it was achieved in November 2020 whereas, in women aged 55–65 years, it was definitively achieved in March 2021.

Differences in participation were also observed by health region (**Supplementary Table 3**). In 2020, screening activity decreased by 24% to 52% depending on the region. In 2021, some regions showed no statistically significant decrease in expected activity while others only experienced a minor reduction (<5%). However, in Girona, where the resumption of activity occurred on January 2022, 44% fewer women got screened.

### Abnormal cytology prevalence

The expected abnormal cytology prevalence in women aged 25–65 for 2020 was 4.40%, but the observed prevalence was 5.92% (ratio=1.35; range=1.22,1.50) (**Table 2**). In 2021, abnormal cytology prevalence was slightly higher than in 2020 (ratio=1.38; range=1.16,1.71).

**Table 2:** Prevalence ratio between observed and expected abnormal cytology (ASCUS+) prevalences by age group in 2020–2021.

Age group	2020				2021			
	Citologies performed (n)	Expected ASCUS+ prevalence (%)	Observed ASCUS+ prevalence (%)	Prevalence ratio (range)	Citologies performed (n)	Expected ASCUS+ prevalence (%)	Observed ASCUS+ prevalence (%)	Prevalence ratio (range)
**25–34**	22,603	7.45	8.55	1.15 (1.06,1.25)	33,457	7.66	9.84	1.29 (1.08,1.59)
**35–44**	24,071	4.12	5.93	1.44 (1.15,1.93)	36,008	4.46	6.35	1.43 (1.06,2.17)
**45–54**	19,606	3.47	4.97	1.43 (1.19,0.78)	31,884	3.69	5.49	1.49 (1.15,2.11)
**55–65**	12,522	1.78	2.65	1.49 (1.18,2.02)	21,225	2.12	3.15	1.49 (1.20,1.96)
**25–65**	78,802	4.40	5.92	1.35 (1.22,1.50)	122,574	4.73	6.53	1.38 (1.16,1.71)

The range shows the prevalence ratios of the observed prevalence versus the 95% prediction interval of the expected prevalence.

By age group, abnormal cytology prevalence ranged between 8.6% in women aged 25–34 to 2.7% in those aged 55–65 in 2020 and between 9.8 and 3.1% in 2021, respectively (**Table 2**). These observed annual abnormal cytology prevalences were between 1.1 and 1.5 times higher than expected. Lower increases in those prevalences were observed in women aged 25–34.

### Time since last normal screening cytology (screening interval)

**Figure 2:**
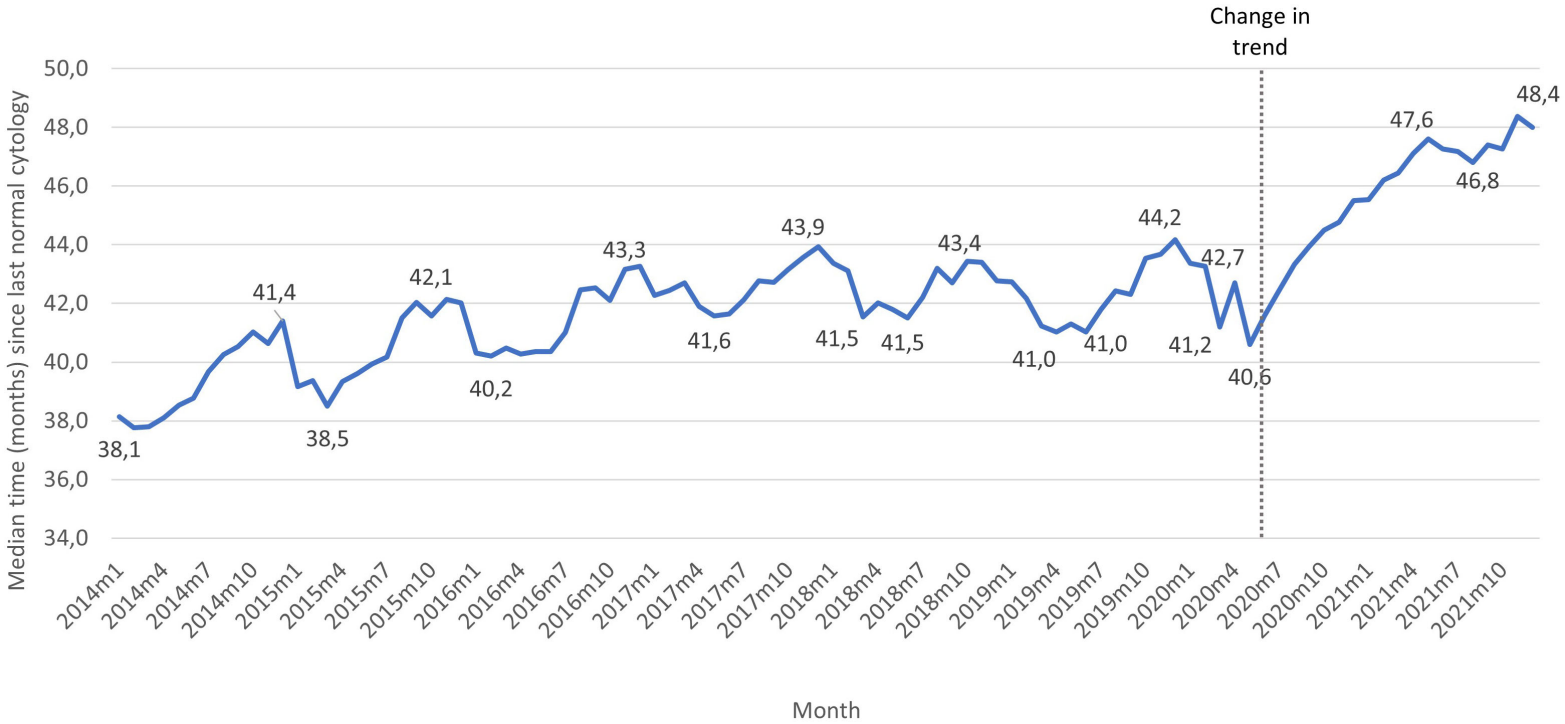
Median time (months) since the last normal screening cytology among women aged 25-65 years with no previous history of abnormal results screened between 2014 and 2021. The figure shows the highest and lower values in median time over the years 2014 and 2021. All monthly values can be found in **Supplementary Table 3**.

Analysis of the trend in the median time since the last normal screening cytology identified an inflection point in June 2020 (**Figure 2**). Between January 2014 and May 2020, the median time increased from 38.1 to 40.6 months, respectively (0.59 months per year; 95%CI=0.46,0.71). From June 2020 onwards, the median time increased by 3.57 months per year (95%CI=2.42,4.71). This resulted in an increase in the median time from 41.6 months in June 2020 to 48.0 months in December 2021 (**Supplementary Table 4**).

Women with an abnormal cytology result showed statistically significant higher screening intervals than those with a normal cytology (45 versus 43.8 months in 2020 and 48.8 versus 46.6 months in 2021).

## Discussion

In Catalonia, after the lockdown period due to the COVID-19 pandemic, lower participation in cervical cancer opportunistic screening was observed in 2020 and 2021, although it highly improved in 2021. Participation was lower at increasing ages. Since the pandemic, a higher abnormal cytology prevalence and a larger screening interval have also been observed.

During the lockdown period in Catalonia, there was up to a 96% reduction in participation to cervical cancer screening in April 2020. Similarly, others have reported the highest impact in April 2020 with similar estimates (7, 8, 28). However, pooled data from other studies worldwide show the most significant reduced cervical cancer screening participation in March 2020 (5), whereas in Slovenia and Japan, it took place in May 2020 (6, 29). The start and end of lockdown periods had likely played a role when the highest reduction was observed.

In 2020, 39% fewer women (47% if the first two months without contentious measures implemented are excluded from the analysis) participated in cervical cancer screening in Catalonia. Different countries have described lower participation or screening test volumes (2–5). However, the impact estimate highly depends on the reported period, which hampers a straight comparison with others. The impact of COVID-19 in Catalonia over the 12 months period of 2020 was higher than the 23% lower cytology volume reported in Slovenia over a 7-month period in the same year (6) and the 36% lower screening estimated for the United States over a 9-month period, also in 2020 (8). However, it was less severe than the 76% reduction in cytology volume observed in Peru over the same 12-month period (9). Overall, in 2021, 2% fewer women participated in cervical cancer screening because of the reduced participation in January (24% reduction) as the third epidemic wave took place and mobility restrictions were again implemented. However, these mobility restrictions did not prohibit attendance to cancer screening visits. Therefore, this lower participation might be related to changes in women’s attitude towards self-care behavior, such as the self-reported lower willingness to attend cervical cancer screening and the perception that screening tests were not urgent in the United Kingdom (30, 31) or the observed longer time to help-seeking for cancer symptoms in Spain (32) after the first COVID-19 pandemic wave. Except for January, observed participation in 2021 was not different than expected, as opposed to the lower estimates observed in Germany and the United States (19, 20).

At an increasing age, Catalonia observed a more considerable impact in screening participation and a slower recovery in regular participation. Lower participation at increasing age was also observed in Slovenia (6), whereas a larger impact was observed in England at young ages (33). The higher impact in women aged 55–65 years than those younger might be related to a higher self-perceived risk of getting severe COVID-19 if infected by the SARS-CoV2 virus at older ages and willingness to avoid contact with the health system unless symptomatic.

The COVID-19 pandemic in Catalonia has not only affected the still opportunistic cervical cancer screening activities. Lower participation in organized cancer screening programs in the region has also been observed for breast (34) and colorectal (35) cancer screening programs. A lower number of diagnoses have also been observed in other chronic diseases managed at primary care services, such as asthma or ischemic heart disease (36).

A higher prevalence of abnormal cytology was observed after the lockdown period (up to 1.5 times higher than expected). The validation of the predictive models with 2014 - 2018 data showed an increasing trend in abnormal cytologies prevalence before the pandemic, yet it was used to adjust the expected prevalences for 2020 and 2021. It follows that the previously observed trend does not explain the observed higher prevalence of abnormal cytology during the pandemic. In Brazil, the prevalence of abnormal cytology findings remained unchanged in the years 2020 and 2021 but, in São Paulo city, the CIN3 prevalence increased (4.5% in 2020 and 3.8% in 2021) in comparison with the 1.9% CIN3 prevalence in 2019 (22). The authors argue that the increase in CIN3 might be related to the follow-up of 2019 abnormal cytology results. Ours is the first study to measure an increase in cytology abnormal prevalence since the pandemic started although we cannot assess whether this increase in cytology abnormality has resulted in an increase in CIN2+ prevalence.

Another potential explanation for the higher prevalence could be related to a larger screening interval. The longer the period after a normal screening result, the lower assurance that the patient remains disease-free and, therefore, a greater prevalence of abnormal cytology results. In our study, we have observed an increasing median time since the last normal cytology from June 2020 onwards among all women and a longer interval in those with an abnormal cytology result compared to those with a normal one. To our knowledge, only one study has explored the cervical cancer screening interval with contrasting findings to our study. In Belgium, no differences in the screening interval were observed between the pre-pandemic and pandemic periods (21). The authors attribute this to the opportunistic screening activity and the follow-up tests done during the lockdown period. In contrast, we excluded follow-up tests from the analyses to accurately estimate the median time since the last screening test, which was 48 months by December 2021.

According to modeling studies, COVID-19-related screening delays have a lower impact on the lifetime cervical cancer risk than the screening test used and the screening frequency (37). Another study estimated that the risk of cervical cancer is higher in women missing the screening round, i.e., those being screened, although with a longer screening interval. Therefore, catch-up campaigns using HPV testing have been suggested to address this issue (38). However, due to a potentially limited capacity in screening organizations, others have estimated that the best balance between screening capacity and health impact is to ensure that women are screened, even if it is done at a longer interval (39). This is consistent with the increased risk observed in women that missed their round (i.e., a delay in screening participation equal to the screening interval) than those that were screened at a longer screening interval (40). However, in Catalonia, cervical cancer screening is still opportunistic and, as a result, women at higher risk (longer time since their last screening) are not identified and offered to screen. Therefore, we are currently piloting the implementation of self-sampling methods in an organized cervical cancer screening program aiming to increase screening capacity and to offer screening to those under screened. However, its scale-up has been slowed down.

In this study, we highlight using predictive models to estimate the expected participation in 2020 after validating our model with expected versus observed data in 2019 instead of assuming constant participation as in previous years. It is also noteworthy to mention that using the SISAP database facilitates the reporting of objective data (others have reported data facilitated by questionnaires to health managers) known to be highly representative of the whole Catalan population. Despite the strengths, our study has some limitations that need to be mentioned. Although changes in participation, abnormal cytology prevalence and screening interval coincide with the COVID-19 pandemic, we cannot rule out other reasons for such changes due to the use of ecological data to study the potential association between our findings and the COVID pandemic. One of these reasons could be that more women use private health services during the pandemic, so participation has been maintained in the overall population but decreased in the public health sector. However, our analysis database only contains data from the latter. On the other hand, the lack of access to structured histology data has prevented us from exploring whether the increase in abnormal cytology prevalence has been translated in an increase in CIN2+ prevalence.

The COVID-19 pandemic has significantly impacted cervical cancer screening in Catalonia, resulting in decreased screening participation, longer screening intervals and a potentially related increase in abnormal cytology prevalence. Therefore, close monitoring of the potential increases in cervical cancer diagnosis or the changes in cancer stage at diagnosis is warranted. Furthermore, to prevent a further increase in advanced cervical cancer diagnosis, it is crucial to expedite the implementation of the organized HPV-based screening program in Catalonia.

## Data availability statement

The data analyzed in this study is subject to the following licenses/restrictions: It contains health records. Requests to access these datasets should be directed to crobles@iconcologia.net.

## Ethics statement

The studies involving humans were approved by Ethics Committee from the Hospital Universitari de Bellvitge. The studies were conducted in accordance with the local legislation and institutional requirements. Written informed consent for participation was not required from the participants or the participants’ legal guardians/next of kin in accordance with the national legislation and institutional requirements.

## Author contributions

CR: Conceptualization, Data curation, Formal analysis, Funding acquisition, Investigation, Methodology, Project administration, Writing – original draft, Writing – review & editing. LM: Conceptualization, Data curation, Formal analysis, Investigation, Methodology, Writing – original draft, Writing – review & editing. RI: Conceptualization, Data curation, Formal analysis, Funding acquisition, Investigation, Methodology, Project administration, Writing – review & editing. ER: Conceptualization, Data curation, Investigation, Methodology, Writing – review & editing. RF: Data curation, Resources, Writing – review & editing. PP-T: Methodology, Writing – review & editing. MB: Methodology, Writing – review & editing. CM-B: Resources, Writing – review & editing. SS: Methodology, Writing – review & editing. JE: Data curation, Resources, Writing – review & editing. LB: Conceptualization, Data curation, Funding acquisition, Investigation, Methodology, Project administration, Resources, Supervision, Writing – review & editing.
