# Supplementary material for: Impact of the COVID-19 pandemic on cervical cancer screening participation, abnormal cytology prevalence and screening interval in Catalonia
Source: Front Oncol. 2024 May 29;14:1338859. doi: 10.3389/fonc.2024.1338859 (PMC11171128; doi:10.3389/fonc.2024.1338859)
Supplement: Supplementary file 1 [file DataSheet_1.docx]

**Supplementary material 1. Additional details on the model validation and selection**

**PARTICIPATION RATE**

Data decomposition of the period 2014-2019 in the overall population:


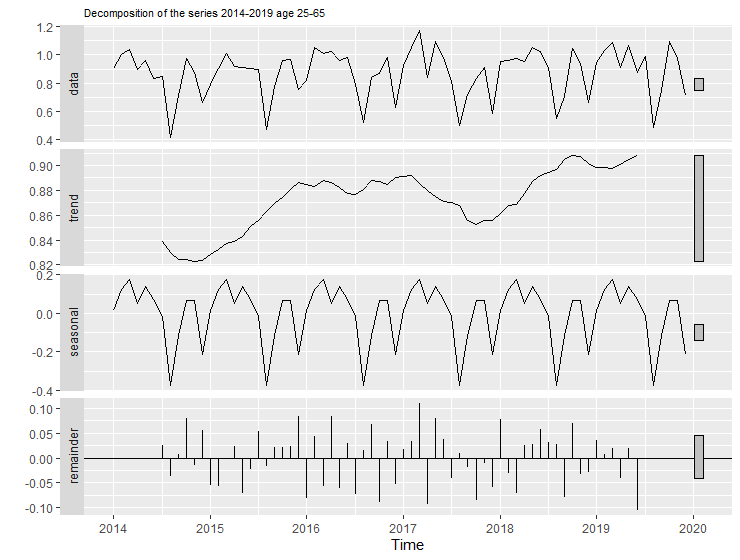


Performance metrics of the training set model (2014-2018) in the test set (2019):

|  | RMSE | MAE | MAPE | MASE |
| --- | --- | --- | --- | --- |
| TSLM with trend and seasonality | 0.0608 | 0.0473 | 5.35005 | 0.6499 |
| Holt-Winters | 0.0610 | 0.0470 | 5.5602 | 0.6465 |
| ARIMA | 0.0727 | 0.0581 | 6.1514 | 0.7991 |

RMSE: Root Mean Squared Error; MAE: Mean Absolute Error; MAPE: Mean Absolute Percentage Error; MASE: Mean Absolute Scaled Error.

Residuals examination from the three predictive models explored:


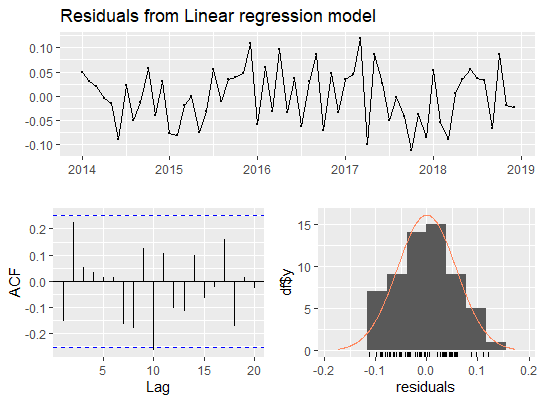

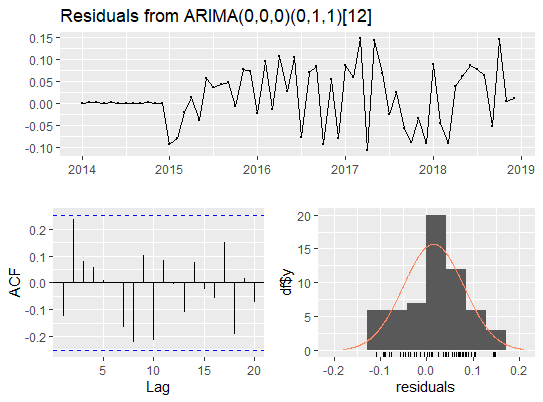


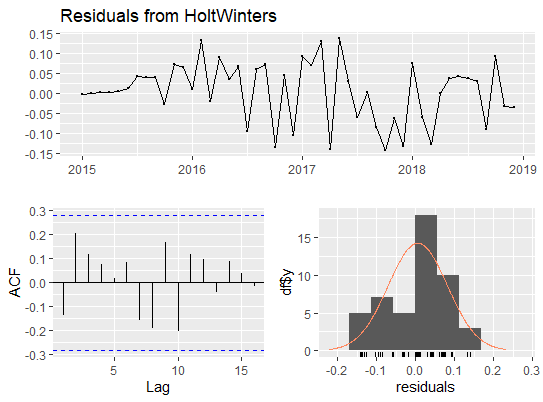


**ABNORMAL CYTOLOGY PREVALENCE**

Data decomposition of the period 2014-2019 in the overall population:


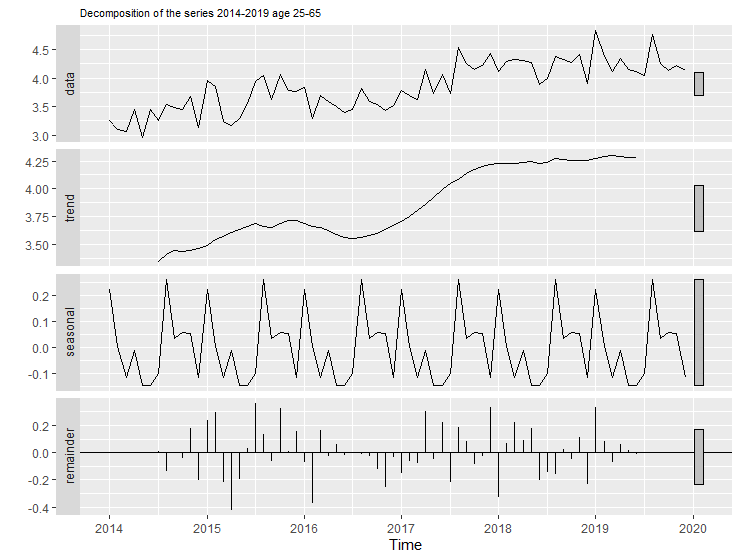


Performance metrics of the training set model (2014-2018) in the test set (2019):

|  | RMSE | MAE | MAPE | MASE |
| --- | --- | --- | --- | --- |
| Holt-Winters | 0.2527 | 0.1957 | 4.5043 | 0.5010 |
| TSLM with trend and seasonality | 0.2393 | 0.2018 | 4.7197 | 0.5167 |
| ARIMA | 0.2799 | 0.2433 | 5.5942 | 0.6230 |

RMSE: Root Mean Squared Error; MAE: Mean Absolute Error; MAPE: Mean Absolute Percentage Error; MASE: Mean Absolute Scaled Error.

Residuals examination from the three predictive models explored:


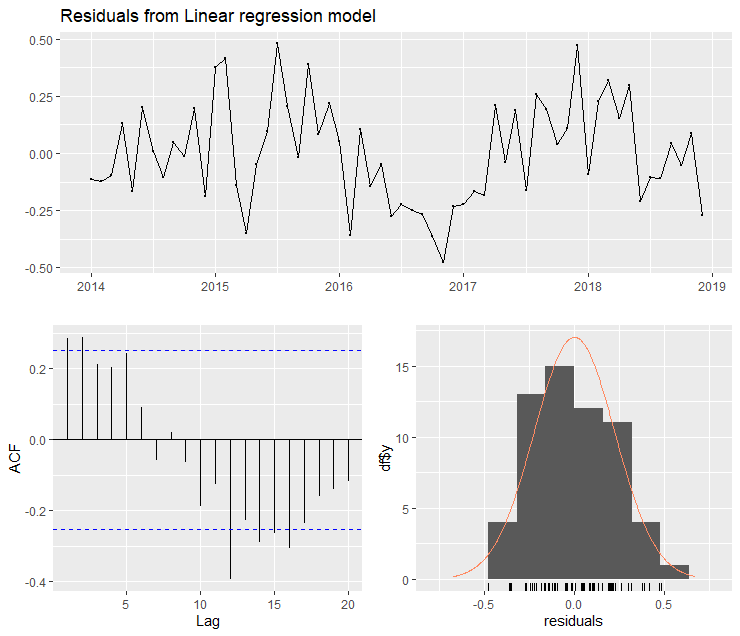

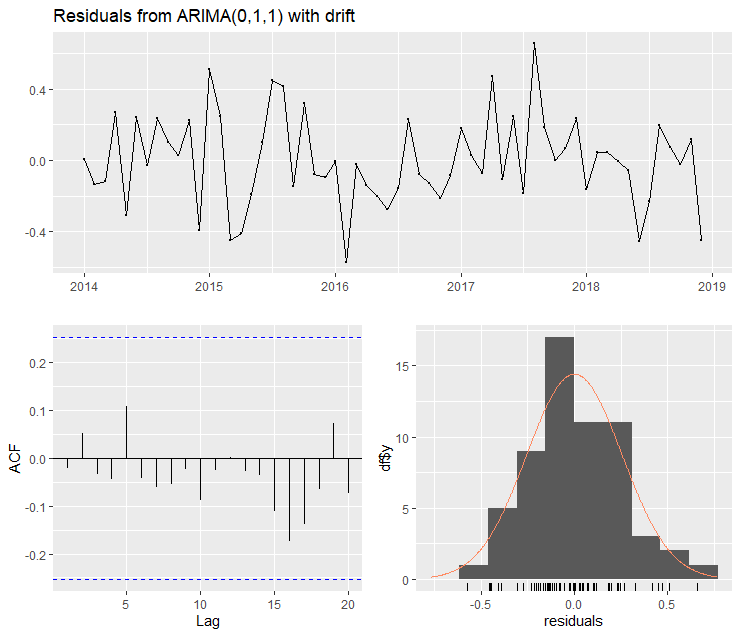


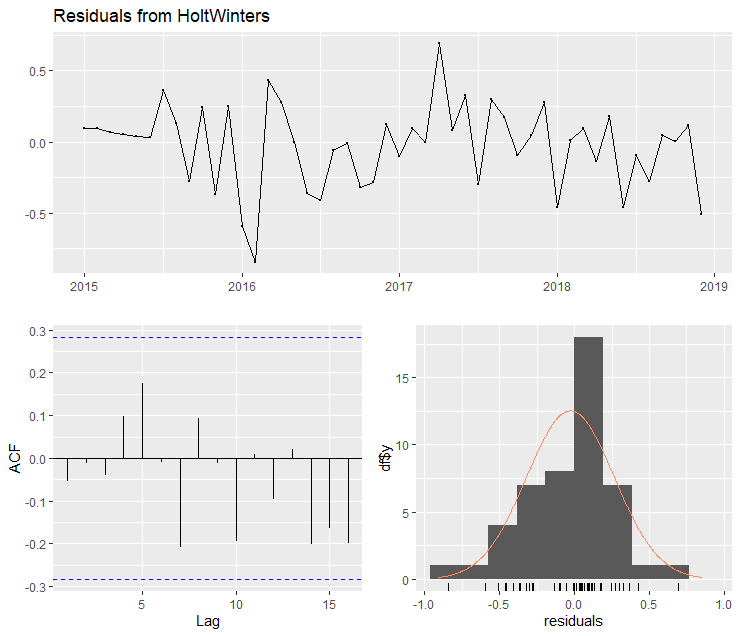


**Supplementary Table 1. Monthly participation ratio between observed and expected screening participation in in 2020-2021**

|  | **25-65** | | | |
| --- | --- | --- | --- | --- |
| **Month** | **N** | **Expected participation (%)** | **Observed participation (%)** | **Rate ratio (range)** |
| **01/2020** | 1,371,907 | 0.94 | 0.94 | n.s. |
| **02/2020** | 1,371,695 | 1.04 | 1.05 | n.s. |
| **03/2020** | 1,371,481 | 1.09 | 0.50 | 0.46 (0.41,0.52) |
| **04/2020** | 1,371,249 | 0.97 | 0.04 | 0.04 (0.03,0.05) |
| **05/2020** | 1,371,145 | 1.05 | 0.10 | 0.09 (0.08,0.11) |
| **06/2020** | 1,371,192 | 0.98 | 0.35 | 0.36 (0.32,0.42) |
| **07/2020** | 1,371,450 | 0.92 | 0.51 | 0.55 (0.48,0.65) |
| **08/2020** | 1,371,566 | 0.53 | 0.28 | 0.53 (0.42,0.71) |
| **09/2020** | 1,371,688 | 0.79 | 0.51 | 0.64 (0.55,0.78) |
| **10/2020** | 1,371,655 | 1.01 | 0.83 | 0.82 (0.72,0.95) |
| **11/2020** | 1,371,770 | 0.99 | 0.92 | n.s. |
| **12/2020** | 1,371,784 | 0.71 | 0.68 | n.s. |
| **01/2021** | 1,371,428 | 0.95 | 0.72 | 0.76 (0.66,0.89) |
| **02/2021** | 1,371,184 | 1.06 | 0.97 | n.s. |
| **03/2021** | 1,370,797 | 1.11 | 1.17 | n.s. |
| **04/2021** | 1,370,579 | 0.98 | 0.98 | n.s. |
| **05/2021** | 1,370,381 | 1.07 | 1.03 | n.s. |
| **06/2021** | 1,370,228 | 0.99 | 0.99 | n.s. |
| **07/2021** | 1,370,135 | 0.93 | 0.80 | n.s. |
| **08/2021** | 1,370,097 | 0.55 | 0.48 | n.s. |
| **09/2021** | 1,370,049 | 0.81 | 0.77 | n.s. |
| **10/2021** | 1,370,030 | 1.02 | 0.90 | n.s. |
| **11/2021** | 1,370,013 | 1.00 | 1.04 | n.s. |
| **12/2021** | 1,370,085 | 0.73 | 0.70 | n.s. |

Footnote: The range shows the proportional change of the observed participation versus the 95% prediction interval of the expected participation.

n.s. – non significant

**Supplementary Table 2. Monthly participation ratio between observed and expected screening participation rates by age group in 2020-2021**

|  | **25-34** | | | | **35-44** | | | | **45-54** | | | | **55-65** | | | |
| --- | --- | --- | --- | --- | --- | --- | --- | --- | --- | --- | --- | --- | --- | --- | --- | --- |
| **Month** | **N** | **Exp. (%)** | **Obs. (%)** | **Rate ratio (range)** | **N** | **Exp. (%)** | **Obs. (%)** | **Rate ratio (range)** | **N** | **Exp. (%)** | **Obs. (%)** | **Rate ratio (range)** | **N** | **Exp. (%)** | **Obs. (%)** | **Rate ratio (range)** |
| **01/2020** | 288401 | 1.20 | 1.21 | n.s. | 379319 | 1.02 | 1.01 | n.s. | 375222 | 0.86 | 0.85 | n.s. | 328965 | 0.68 | 0.71 | n.s. |
| **02/2020** | 287968 | 1.26 | 1.28 | n.s. | 378543 | 1.14 | 1.13 | n.s. | 375658 | 0.99 | 1.02 | n.s. | 329526 | 0.80 | 0.79 | n.s. |
| **03/2020** | 287293 | 1.29 | 0.61 | 0.47 (0.42,0.54) | 377844 | 1.20 | 0.54 | 0.45 (0.40,0.52) | 376200 | 1.05 | 0.48 | 0.45 (0.4,0.53) | 330144 | 0.84 | 0.38 | 0.45 (0.40,0.53) |
| **04/2020** | 286669 | 1.17 | 0.06 | 0.06 (0.05,0.06) | 377042 | 1.06 | 0.05 | 0.04 (0.04,0.05) | 376758 | 0.93 | 0.02 | 0.03 (0.02,0.03) | 330780 | 0.74 | 0.02 | 0.03 (0.03,0.04) |
| **05/2020** | 285951 | 1.23 | 0.15 | 0.12 (0.11,0.14) | 376153 | 1.15 | 0.12 | 0.10 (0.09,0.12) | 377630 | 1.02 | 0.09 | 0.09 (0.08,0.1) | 331411 | 0.83 | 0.04 | 0.05 (0.04,0.06) |
| **06/2020** | 285451 | 1.15 | 0.47 | 0.41 (0.36,0.48) | 375325 | 1.06 | 0.42 | 0.40 (0.35,0.47) | 378278 | 0.94 | 0.31 | 0.33 (0.28,0.39) | 332138 | 0.77 | 0.22 | 0.29 (0.25,0.34) |
| **07/2020** | 285072 | 1.12 | 0.67 | 0.60 (0.52,0.70) | 374179 | 1.00 | 0.59 | 0.59 (0.51,0.70) | 379295 | 0.89 | 0.45 | 0.50 (0.43,0.61) | 332904 | 0.69 | 0.33 | 0.48 (0.41,0.58) |
| **08/2020** | 284753 | 0.78 | 0.43 | 0.56 (0.46,0.71) | 373254 | 0.60 | 0.32 | 0.54 (0.43,0.73) | 379931 | 0.46 | 0.23 | 0.51 (0.38,0.75) | 333628 | 0.32 | 0.16 | 0.49 (0.36,0.77) |
| **09/2020** | 284321 | 1.02 | 0.72 | 0.70 (0.61,0.83) | 372451 | 0.87 | 0.59 | 0.67 (0.57,0.81) | 380335 | 0.74 | 0.45 | 0.62 (0.51,0.78) | 334581 | 0.57 | 0.31 | 0.55 (0.46,0.69) |
| **10/2020** | 283899 | 1.23 | 1.10 | n.s. | 371482 | 1.10 | 0.92 | 0.84 (0.74,0.98) | 381005 | 0.94 | 0.76 | 0.81 (0.69,0.96) | 335269 | 0.78 | 0.56 | 0.73 (0.63,0.85) |
| **11/2020** | 283548 | 1.21 | 1.23 | n.s. | 370652 | 1.10 | 1.04 | n.s. | 381620 | 0.91 | 0.85 | n.s. | 335950 | 0.74 | 0.59 | 0.79 (0.68,0.94) |
| **12/2020** | 283204 | 0.95 | 0.96 | n.s. | 369715 | 0.80 | 0.79 | n.s. | 382352 | 0.63 | 0.61 | n.s. | 336513 | 0.49 | 0.41 | n.s. |
| **01/2021** | 282777 | 1.24 | 1.03 | 0.83 (0.74,0.96) | 368511 | 1.04 | 0.82 | 0.79 (0.68,0.92) | 382972 | 0.86 | 0.63 | 0.74 (0.62,0.9) | 337168 | 0.67 | 0.44 | 0.65 (0.55,0.79) |
| **02/2021** | 282394 | 1.30 | 1.30 | n.s. | 367692 | 1.16 | 1.09 | n.s. | 383650 | 0.98 | 0.89 | n.s. | 337448 | 0.80 | 0.63 | 0.80 (0.69,0.93) |
| **03/2021** | 281919 | 1.33 | 1.49 | n.s. | 366751 | 1.22 | 1.30 | n.s. | 384122 | 1.05 | 1.09 | n.s. | 338005 | 0.84 | 0.86 | n.s. |
| **04/2021** | 281432 | 1.21 | 1.24 | n.s. | 365899 | 1.08 | 1.09 | n.s. | 384577 | 0.93 | 0.91 | n.s. | 338671 | 0.74 | 0.73 | n.s. |
| **05/2021** | 280839 | 1.27 | 1.25 | n.s. | 364989 | 1.16 | 1.14 | n.s. | 385174 | 1.02 | 1.01 | n.s. | 339379 | 0.83 | 0.75 | n.s. |
| **06/2021** | 280488 | 1.19 | 1.23 | n.s. | 363970 | 1.08 | 1.08 | n.s. | 385730 | 0.94 | 0.96 | n.s. | 340040 | 0.77 | 0.73 | n.s. |
| **07/2021** | 279919 | 1.16 | 0.98 | 0.84 (0.74,0.98) | 363133 | 1.02 | 0.90 | n.s. | 386286 | 0.88 | 0.78 | n.s. | 340797 | 0.69 | 0.57 | n.s. |
| **08/2021** | 279465 | 0.82 | 0.71 | n.s. | 362009 | 0.62 | 0.54 | n.s. | 386992 | 0.46 | 0.43 | n.s. | 341631 | 0.32 | 0.30 | n.s. |
| **09/2021** | 278835 | 1.07 | 1.00 | n.s. | 361136 | 0.89 | 0.87 | n.s. | 387539 | 0.73 | 0.72 | n.s. | 342539 | 0.56 | 0.54 | n.s. |
| **10/2021** | 278439 | 1.28 | 1.12 | n.s. | 360197 | 1.12 | 0.99 | n.s. | 388154 | 0.94 | 0.85 | n.s. | 343240 | 0.77 | 0.69 | n.s. |
| **11/2021** | 277933 | 1.25 | 1.23 | n.s. | 359369 | 1.12 | 1.15 | n.s. | 388760 | 0.91 | 1.01 | n.s. | 343951 | 0.74 | 0.82 | n.s. |
| **12/2021** | 277512 | 0.99 | 0.90 | n.s. | 358515 | 0.82 | 0.77 | n.s. | 389233 | 0.63 | 0.66 | n.s. | 344825 | 0.49 | 0.52 | n.s. |

Footnote: The range shows the proportional change of the observed prevalence versus the 95% prediction interval of the expected prevalence.

n.s. – non significant

**Supplementary Table 3. Participation rate between observed and expected screening participation of women aged 25-65 years in Catalonia by health region in 2020-2021**

|  |  | **2020** | | |  | **2021** | | |
| --- | --- | --- | --- | --- | --- | --- | --- | --- |
| **Health region** | **N** | **Expected participation (%)** | **Observed participation (%)** | **Participation ratio (range)** | **N** | **Expected participation (%)** | **Observed participation (%)** | **Participation ratio (range)** |
| **All regions except Girona** | 1,371,549 | 10.94 | 6.70 | 0.61 (0.56,0.68) | 1,370,417 | 10.79 | 10.56 | 0.98 (0.97,0.99) |
| **Lleida** | 93,234 | 9.21 | 5.62 | 0.61 (0.54,0.70) | 93,171 | 9.39 | 9.39 | n.s. |
| **Tarragona** | 94,915 | 10.27 | 7.74 | 0.75 (0.71,0.80) | 94,812 | 10.74 | 10.51 | 0.98 (0.96,0.99) |
| **Barcelona** | 273,633 | 9.39 | 5.69 | 0.61 (0.54,0.68) | 274,548 | 9.31 | 9.15 | 0.98 (0.97,0.99) |
| **Girona** | 47,393 | 14.69 | 7.07 | 0.48 (0.38,0.65) | 47,476 | 15.28 | 8.53 | 0.56 (0.43,0.81) |
| **Metropolitana Sud** | 359,269 | 11.26 | 6.25 | 0.55 (0.49,0.63) | 358,717 | 11.41 | 10.80 | 0.95 (0.91,0.99) |
| **Metropolitana Nord** | 390,175 | 12.31 | 7.53 | 0.61 (0.55,0.69) | 389,432 | 11.07 | 11.64 | n.s. |
| **Catalunya Central** | 115,071 | 10.94 | 7.65 | 0.70 (0.64,0.77) | 114,754 | 11.07 | 10.86 | 0.98 (0.97,0.99) |
| **Alt Pirineu-Aran** | 11,750 | 11.26 | 8.59 | 0.76 (0.68,0.87) | 11,683 | 12.76 | 12.76 | n.s. |
| **Terres Ebre** | 33,502 | 8.17 | 6.06 | 0.74 (0.65,0.86) | 33,300 | 8.52 | 8.52 | n.s. |

Footnote: The range shows the proportional change of the observed participation versus the 95% prediction interval of the expected participation.

n.s. – non significant

**Supplementary Table 4. Median time (months) since the last normal screening cytology among women aged 25-65 years with no previous history of abnormal results screened for each month between 2014 and 2021**

|  | **Median time since last normal cytology (months)** | | | | | | | |
| --- | --- | --- | --- | --- | --- | --- | --- | --- |
|  | **2014** | **2015** | **2016** | **2017** | **2018** | **2019** | **2020** | **2021** |
| **January** | 38.1 | 39.2 | 40.3 | 42.3 | 43.4 | 42.7 | 43.4 | 45.5 |
| **February** | 37.8 | 39.4 | 40.2 | 42.5 | 43.1 | 42.2 | 43.3 | 46.2 |
| **March** | 37.8 | 38.5 | 40.5 | 42.7 | 41.5 | 41.2 | 41.2 | 46.4 |
| **April** | 38.1 | 39.3 | 40.3 | 41.9 | 42.0 | 41.0 | 42.7 | 47.1 |
| **May** | 38.5 | 39.6 | 40.4 | 41.6 | 41.8 | 41.3 | 40.6 | 47.6 |
| **June** | 38.8 | 39.9 | 40.4 | 41.6 | 41.5 | 41.0 | 41.6 | 47.3 |
| **July** | 39.7 | 40.2 | 41.0 | 42.1 | 42.2 | 41.8 | 42.5 | 47.2 |
| **August** | 40.3 | 41.5 | 42.5 | 42.8 | 43.2 | 42.4 | 43.3 | 46.8 |
| **September** | 40.5 | 42.0 | 42.5 | 42.7 | 42.7 | 42.3 | 43.9 | 47.4 |
| **October** | 41.0 | 41.6 | 42.1 | 43.2 | 43.4 | 43.5 | 44.5 | 47.3 |
| **November** | 40.6 | 42.1 | 43.2 | 43.6 | 43.4 | 43.7 | 44.8 | 48.4 |
| **December** | 41.4 | 42.0 | 43.3 | 43.9 | 42.8 | 44.2 | 45.5 | 48.0 |

Footnote: The cell shaded in grey depicts the change in the screening interval trend
